# Movement of environmental threats modifies the relevance of the defensive eye-blink in a spatially-tuned manner

**DOI:** 10.1038/s41598-019-40075-x

**Published:** 2019-03-06

**Authors:** R. Somervail, R. J. Bufacchi, Y. Guo, M. Kilintari, G. Novembre, D. Swapp, A. Steed, G. D. Iannetti

**Affiliations:** 1 Department of Neuroscience, Physiology and Pharmacology, University College London (UCL), London, UK; 2 Neuroscience and Behaviour Laboratory, Istituto Italiano di Tecnologia (IIT), Rome, Italy; 3 Department of Computer Science, University College London (UCL), London, UK

## Abstract

Subcortical reflexive motor responses are under continuous cortical control to produce the most effective behaviour. For example, the excitability of brainstem circuitry subserving the defensive hand-blink reflex (HBR), a response elicited by intense somatosensory stimuli to the wrist, depends on a number of properties of the eliciting stimulus. These include face-hand proximity, which has allowed the description of an HBR response field around the face (commonly referred to as a defensive peripersonal space, DPPS), as well as stimulus movement and probability of stimulus occurrence. However, the effect of *stimulus-independent* movements of objects in the environment has not been explored. Here we used virtual reality to test whether and how the HBR-derived DPPS is affected by the presence and movement of threatening objects in the environment. In two experiments conducted on 40 healthy volunteers, we observed that threatening arrows flying towards the participant result in DPPS expansion, an effect directionally-tuned towards the source of the arrows. These results indicate that the excitability of brainstem circuitry subserving the HBR is continuously adjusted, taking into account the movement of environmental objects. Such adjustments fit in a framework where the relevance of defensive actions is continually evaluated, to maximise their survival value.

## Introduction

To survive in a fast-changing environment, animals must detect and react appropriately to unexpected events. Subcortical reflex circuits allow the execution of fast motor responses. However, stereotyped reflex responses are not always optimal to ensure survival. Therefore, cortical mechanisms can top-down modulate subcortical reflex circuits to produce more appropriate motor reactions, taking into account many *stimulus-related* factors^1–5^. One important factor is the proximity of stimuli with respect to the body. Indeed, sudden stimuli occurring close to the body or specific body parts often elicit stronger defensive reactions^6–9^.

A typical example of such a proximity-dependent modulation is the enhancement of the blink reflex elicited by intense somatosensory stimulation of the median nerve at the wrist (hand-blink reflex; HBR). Although the HBR is mediated by a subcortical circuit in the brainstem^10,11^, its magnitude is increased when the stimulated hand is closer to the face^8,12^. We have recently used geometric modelling to characterise the HBR response field following somatosensory stimulation in a large number of hand positions, and thus derived its fine-grained spatial features around the face^13,14^. The HBR response field indicates the behavioural relevance of blinking as a function of stimulus position. This field is commonly referred to as an instance of a peripersonal space (PPS)^15^. Many peripersonal response fields are affected by stimulus properties other than proximity^15–17^, and this HBR-derived defensive PPS (DPPS) is no exception: stimulus energy, inter-stimulus interval and probability of stimulus occurrence are all positively related to HBR magnitude^8,10,12^; similarly, movement of the stimulated hand towards the face expands the HBR-derived DPPS^18–20^.

In contrast, the effect of *stimulus-independent* environmental factors on DPPS is less explored; for the HBR response field, to the best of our knowledge, only two environmental factors have been tested: gravitational cues^21^ and physical barriers between the hand and face^12^. Importantly, both factors are static, and the effect of moving environmental objects that are separate to the stimulus triggering the reflex remains unexplored.

Given the working hypothesis that HBR magnitude should be modulated to produce the most effective defensive behaviour, here we asked whether and how the DPPS is affected by the presence and movement of threatening objects in the environment. To address this question, we performed two experiments in 40 healthy human volunteers. We recorded the HBR elicited by electrical somatosensory stimuli delivered at different hand positions while participants were immersed in virtual reality (VR) environments and exposed to fast-moving virtual arrows originating from several locations. In Experiment 1, we observed that the occurrence of arrows flying towards the participant altered the shape of the HBR proximity-response function, suggesting a more gradual fall-off and a DPPS expansion. In Experiment 2, the arrows originated from spatially distinct sources and we observed that the effect was directionally-tuned towards the source of the arrows.

## Methods

### Participants

40 healthy volunteers participated in the study and gave written informed consent before taking part. Experimental procedures were approved by the University College London ethics committee. All experiments were performed in accordance with the Declaration of Helsinki, as well as local guidelines and regulations. Experiment 1 included 20 participants (11 women; age range 19–41 yr, mean ± SD 24.9 ± 6.0). Experiment 2 included 20 participants (12 women; age range 18–25 yr, mean ± SD 20.8 ± 2.1).

### Somatosensory Stimulation

Somatosensory stimuli consisted of constant-current, 200-µs long square pulses generated by an electrical stimulator (DS7A, Digitimer). Stimuli were delivered using a surface bipolar electrode placed on the median nerve at the wrist. In Experiment, 1 stimuli were delivered to the right hand; In Experiment 2, stimuli were delivered to either hand. Stimulus intensity was adjusted in each participant at the beginning of each recording block, to elicit a clear HBR in three consecutive trials^8,10^. Participants who refused an increase in stimulus intensity before three clear HBR responses were observed, or who had a clear HBR in less than 50% of trials were considered non-responders and did not take part in the experiment^12^. In HBR responders the mean stimulus intensity across participants (±SD) was 39.4 ± 15.0 mA [experiment 1], and 46.8 ± 21.3 mA [experiment 2]. In Experiment 1, participants were recruited from a group of previously screened HBR responders^8,21^. In Experiment 2, out of 64 participants tested, 20 (i.e. 31%) were classified as HBR-responders and took part in the rest of the experiment. This 31% response rate is lower than commonly reported (~60%^8,10,12,13^), which may have been due to the head mounted display acting as a protective screen for certain participants^12^.

### EMG Recording

EMG activity was recorded from the orbicularis oculi muscle, bilaterally, using pairs of surface electrodes with the active electrode placed over the mid-lower eyelid and the reference electrode a few centimetres laterally to the outer canthus. Signals were amplified and digitized at a sampling rate of 2048 Hz (SD 32, Micromed).

### Virtual Reality

**Figure 1:**
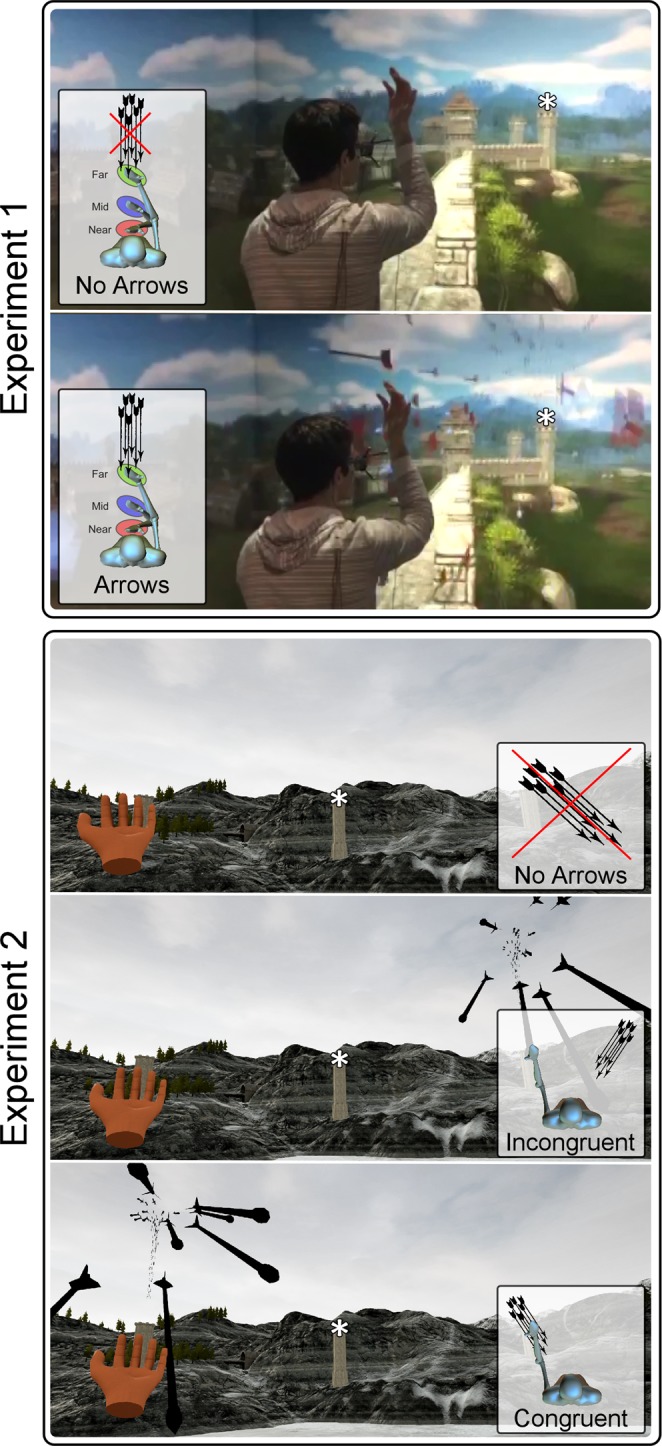
Virtual reality environments and experimental conditions for Experiment 1 and Experiment 2. Each image shows the virtual environment during one experimental condition. Asterisks indicate the fixation points. Insets show top-down schematic views of the experimental conditions. *Experiment 1*. The CAVE virtual reality system is shown with a participant holding their hand in the Middle hand position with either no arrows (top image) or arrows present (bottom image). *Experiment 2*. The display of the HTC Vive headset is shown in three conditions for a block in which the left hand was stimulated. The top image shows the condition with no arrows. The middle image shows the condition with arrows that were launched from the tower on the opposite side to the stimulated hand (spatially incongruent). The bottom image shows the condition in which arrows were launched on the same side to the stimulated hand (spatially congruent).

In both experiments, participants were immersed in virtual reality environments programmed in-house. In Experiment 1, the VR environment was programmed in Unity, and was presented to the participants in the CAVE system at the UCL Computer Science Department (https://vr.cs.ucl.ac.uk). This system offers the advantage of allowing participants to see their entire body within the virtual environments (Fig. 1; *Experiment 1*). In Experiment 2, the VR environment was programmed in Unreal Engine 4 and was presented to the participants through the HTC Vive head-mounted display. In both experiments participants remained seated during the data collection but were permitted to explore the virtual environment between blocks. Given that the head-mounted display prevented participants from seeing their own body, in Experiment 2 the position of each hand was tracked with a motion controller that allowed us to project a virtual hand at the position of the participant’s own hand (Fig. 1; *Experiment 2*). Between each block of Experiment 2, participants played a simple game requiring them to hit target balloons with their virtual hand, since the simultaneous movement of virtual and real body parts has been shown to enhance embodiment^22,23^.

### Experimental Design

In Experiment 1, participants sat facing a virtual tower (Fig. 1; *Experiment 1*). They were instructed to keep their gaze fixed on the top of the tower, with the forearms resting on their legs. Somatosensory stimuli were delivered to the right hand, with an inter-stimulus interval of approximately 30 s. Approximately 10 s before stimulus onset, participants were verbally instructed to place and hold their hand in one of the following three positions. In the ‘Near’ position, the forearm was at ~75 degrees with respect to the arm (with the wrist ~4 cm from the face, on the midline). In the ‘Middle’ position, the forearm was at ~90 degrees with respect to the arm. In the ‘Far’ position, the forearm was extended at ~180 degrees with respect to the arm. In all positions, the hand was aligned between the eyes and the top of the tower. After each stimulus, participants returned to the resting position, with the forearm resting on the thigh. 72 somatosensory stimuli were delivered in three separate blocks. Thus, 8 stimuli were delivered at each hand position in each block. In half of the trials, a cluster of arrows was launched towards the participant’s face from the tower in front of them. In the remaining half of the trials, somatosensory stimuli were delivered without arrows. The order of hand positions and the presence of arrows were pseudorandomised so that no condition occurred more than twice in a row.

In Experiment 2, participants faced three equidistant virtual towers: one on the midline and the other two on the right and left sides, at approximately 33 degrees from the midline. Twenty-four somatosensory stimuli were delivered to each hand, in 8 separate blocks; in half of the blocks, somatosensory stimuli were delivered to the left hand and in the other half they were delivered to the right hand. Each trial started with a visual instruction to keep the gaze on the middle tower. In a third of trials, no arrows were launched (condition ‘No Arrows’). In the remaining two-thirds of the trials, a cluster of arrows was launched towards the participant’s face: in half of *these* trials, arrows were launched from the tower ipsilateral to the stimulated hand (condition ‘Arrows-Congruent’); in the remaining half of these trials, arrows were launched from the tower *contralateral* to the stimulated hand (condition ‘Arrows-Incongruent’). These three conditions were pseudorandomised, with the constraint that no condition occurred more than twice in a row. In each trial with arrows, participants were informed of the source of the arrows by a visual cue displayed above the corresponding tower. In contrast to Experiment 1, the stimulated hand was always kept in the same position: the left hand was aligned between the eyes and the left tower, and the right hand was aligned between the eyes and the right tower. The forearm was kept between ~100 and 130 degrees with respect to the arm. The hand position was adjusted in each individual participant to make sure that (1) the arrows presented in the congruent condition made contact with the hand, while (2) the arrows presented in the incongruent condition did not.

### Data Analysis and Statistics

EMG signals were pre-processed using Letswave 6 (www.letswave.org)^24^. EMG signals were first band-pass filtered between 55 and 395 Hz, notch filtered (width = 2 Hz) at each harmonic of 50 Hz from 100 Hz to 350 Hz, and full-wave rectified. Given the lack of previously-reported interactions between the factor ‘eye side’ (contralateral and ipsilateral to the stimulated hand) and a number of experimental manipulations^8^, HBR responses were averaged between eyes (as in refs^13,14,18,19,25^). HBR magnitude was calculated for each trial as the area under the curve (AUC)^8^. For each participant, AUC values were transformed into Z scores. Normalised AUC values were finally averaged across trials for each experimental condition. In Experiment 1, this procedure yielded 6 average AUC values for each participant: (1) Hand Far, No Arrows; (2) Hand Far, Arrows; (3) Hand Middle, No Arrows; (4) Hand Middle, Arrows; (5) Hand Near, No Arrows; (6) Hand Near, Arrows. In Experiment 2, this procedure yielded 3 AUC average values for each participant: (1) Arrows-Congruent; (2) Arrows-Incongruent; (3) No Arrows. Given that HBR responses elicited by left and right hand stimulation are no different, in Experiment 2 we merged the results from both hands together, as in Sambo *et al*.^8^.

In Experiment 1, we performed a two-way, repeated-measures ANOVA with the within-subject experimental factors ‘*Hand Position’* (three levels: Near, Middle, Far) and ‘*Arrows’* (two levels: Yes, No). In Experiment 2, we performed a one-way, repeated-measures ANOVA with the within-subject experimental factor ‘*Arrows’* (three levels: No Arrows, Arrows-Incongruent, Arrows-Congruent). P values were corrected for violations of the sphericity assumption (Greenhouse-Geisser correction; P_GG_). Significant main effects and interactions were followed up with *post-hoc* t tests. Effect sizes were calculated as Cohen’s d^26^.

### Geometric Modelling

We also performed a geometric modelling analysis, which assumes that when a shock occurs at the wrist, the brain makes an assessment of the probability that the possible threat represented by the shock might interact with - and thus damage - the face^14^. This probability, i.e. the estimated hit-probability of the *stimulus* eliciting the HBR (not of the arrows) is affected by the estimated directions in which the threat might move: if the threat is more likely to move towards the face, the HBR in response to that threat will be stronger. We postulated two nested models. In the first model, the possible directions in which the threat represented by the somatosensory stimulus might move were *not* affected by the movement of the arrows. In the second model, the possible directions of the threat *were* affected by the arrows: the movement bias of the threat represented by the shock on the wrist was altered in the same direction as the arrows were moving. The strength of the bias postulated by the second model was varied in order to find the best fit between the model and the HBR magnitude^14,21^. A small baseline bias towards the face, regardless of the presence and the trajectory of the arrows, was also assumed in both models.

Goodness of fit testing allowed us to assess whether each geometric model fit the data or had to be rejected. For this analysis, the data must be normally distributed and have equal variance. To satisfy these conditions, we calculated the Z-scores of the power-transformed AUCs, as described in detail elsewhere^14^. After these transformations, data from Experiment 1 was normally distributed (p = 0.207; Anderson-Darling test) and had equal variance across all conditions (p = 0.313; Bartlett’s test). However, data from Experiment 2 did not have equal variance (p = 0.0314; Bartlett’s test), and thus goodness of fit testing could not be performed on that data. As such, the parameter fitting and initial assessment of model validity had to be performed on Experiment 1 first. Because one of the models was found to fit the data of Experiment 1 well (see results), we then used that fitted model to calculate the probability that the threat represented by the somatosensory stimulus hits the face for Experiment 2. Subsequently, we performed a linear mixed effects model to test whether these probabilities were good predictors of the HBR magnitudes in Experiment 2. Thus, the linear mixed effects model predicted the trial-by-trial HBR magnitude with a fixed effect of hit probability and a random effect of subject number.

## Results

### Experiment 1

To investigate the effect of arrows on the proximity-dependent modulation of the HBR, we performed a two-way, repeated-measures ANOVA with the within-subject experimental factors ‘*Hand Position’* (three levels: Near, Middle, Far) and ‘*Arrows’* (two levels: Yes, No). We observed strong evidence for a main effect of ‘*Hand Position’* on HBR magnitude (F = 7.69; P_GG_ = 0.00194), and no evidence for a main effect of ‘*Arrows’* (F = 2.17; P_GG_ = 0.157). Importantly, there was evidence for an interaction between the two factors (F = 6.02; P_GG_ = 0.00813; all ANOVA results are summarised in Table 1). Post-hoc t tests revealed that the source of the interaction was (1) a larger HBR in the ‘Middle’ position when arrows were fired than when no arrows were fired, while this was not the case in the ‘Near’ and ‘Far’ positions, and (2) the lack of an effect of hand-position when arrows were fired, while there was a strong effect of hand-position when no arrows were fired (results of post-hoc tests are detailed in Table 2). These results indicate that the shape of the proximity-response function of the HBR changed, suggesting a more gradual fall-off when arrows were fired.

**Table 1:** Experiments 1 & 2 - Summary of ANOVA results.

	F	P_GG_
*Experiment 1*		
**Main effect of ‘Hand Position’**	**7**.**69**	**0**.**00194**
Main effect of ‘Arrows’	2.17	0.15700
**Interaction**	**6**.**02**	**0**.**00813**
*Experiment 2*		
**Effect of ‘Arrows’**	**4**.**91**	**0**.**02370**

**Table 2:** Experiment 1 - Summary of post-hoc tests.

*p values*							
		No Arrows			Arrows		
		Near	Middle	Far	Near	Middle	Far
**No Arrows**	Near	N/A					
	Middle	**7**.**2E-05**	N/A				
	Far	**0**.**001**	0.750	N/A			
**Arrows**	Near	0.759	**0**.**005**	**0**.**012**	N/A		
	Middle	0.977	**0**.**040**	0.063	0.512	N/A	
	Far	0.774	0.078	0.112	0.120	0.528	N/A
***t values***							
**No Arrows**	Near	N/A					
	Middle	**−5**.**045**	N/A				
	Far	**−3**.**727**	0.324	N/A			
**Arrows**	Near	0.311	**3**.**193**	**2**.**775**	N/A		
	Middle	−0.029	**2**.**199**	1.975	−0.668	N/A	
	Far	−0.291	1.864	1.665	−1.629	−0.642	N/A
***d values***							
**No Arrows**	Near	N/A					
	Middle	**−1**.**128**	N/A				
	Far	**−0**.**833**	N/A	N/A			
**Arrows**	Near	N/A	**0**.**714**	**0**.**621**	N/A		
	Middle	N/A	**0**.**492**	N/A	N/A	N/A	
	Far	N/A	N/A	N/A	N/A	N/A	N/A

### Experiment 2

To investigate whether the change in shape of the HBR proximity response function observed in Experiment 1 was sensitive to the trajectory of the arrows, we performed a one-way, repeated-measures ANOVA with the within-subject experimental factor ‘*Arrows’* (three levels: No Arrows, Arrows-Incongruent, Arrows-Congruent). We observed evidence of a difference between experimental conditions (F = 4.91, P_GG_ = 0.0237; Table 1). Post-hoc t tests revealed that the source of this difference was a larger HBR in trials with arrows that were spatially congruent to the stimulated hand, compared to (1) trials with no arrows and (2) trials with arrows that were spatially incongruent. By comparison, there was no difference between trials with spatially incongruent arrows and trials without arrows. Results of post-hoc tests are detailed in Table 3.

**Table 3:** Experiment 2 - Summary of post-hoc tests.

	t values	p values	d values
Arrows-Congruent vs No Arrows	**2**.**27**	**0**.**0352**	**0**.**507**
Arrows-Congruent vs Arrows-Incongruent	**3**.**36**	**0**.**0033**	**0**.**751**
Arrows-Incongruent vs No Arrows	0.377	0.7110	N/A

### Geometric Modelling

The geometric model of HBR-derived DPPS assumes that when a shock occurs at the wrist, the brain assesses the probability that the possible threat - represented by this shock - might interact with and thus damage the face. This probability is affected by the estimated directions in which the threat might move. The model in which these estimated directions were *not* affected by the movement of the arrows was clearly rejected by the goodness of fit testing on the data from Experiment 1 (GoF p = 0.00195; note that for this type of test, p < 0.05 indicates that the model is rejected). In contrast, the model in which these directions *were* affected by movement of the arrows fit the data from Experiment 1 well (GoF p = 0.382; note that p > 0.05 indicates that the model is accepted).

The linear mixed effects model showed that the geometric model that fit the data well in Experiment 1 was also a significant predictor of HBR magnitude in Experiment 2 (p = 0.00216; note that p values for this type of test should be interpreted the usual way). Thus, these modelling results provide support for the notion that movements of environmental objects (separate from those eliciting the defensive reflex) can affect the brain’s assessment of the relevance of defensive actions, by influencing the predicted probability of contact of the reflex-eliciting stimulus.

## Discussion

**Figure 2:**
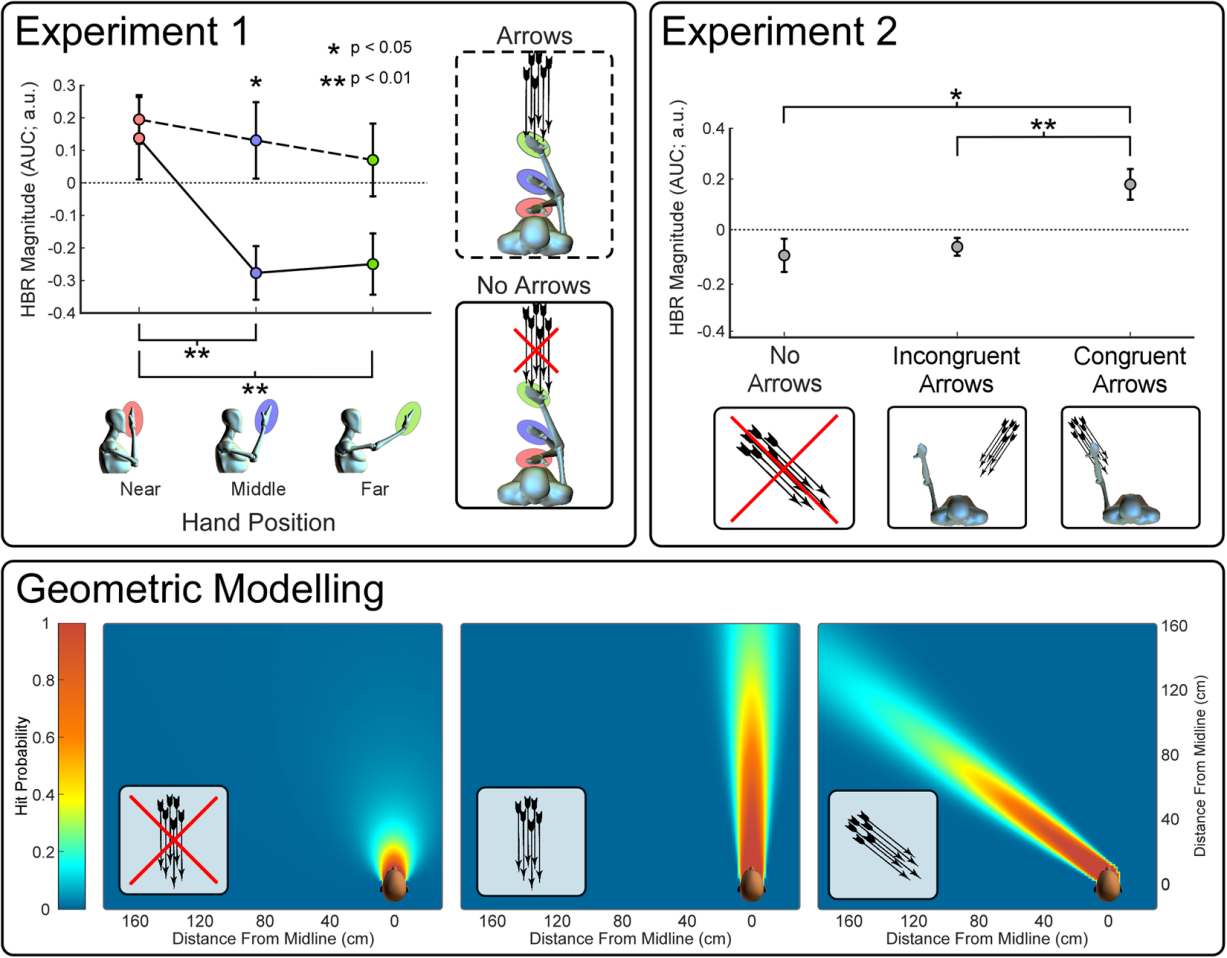
Experimental results and model-fitting. Asterisks show significant comparisons between conditions (post-hoc t tests). Error bars show SEM for each condition. *Top-left panel: Experiment 1*. Mean HBR magnitude (area under the curve; AUC) across all participants for each condition of Experiment 1. The solid line shows the proximity response function with no arrows present, while the dashed line shows the response function with arrows present. The shape of this function changed when arrows were fired, suggesting a more gradual fall-off. The HBR was larger when arrows were fired in the Middle hand position than when no arrows were fired. Differences between hand positions were found only when arrows were not fired. *Top-right panel: Experiment 2*. Mean HBR magnitude (AUC) across all participants for each condition of Experiment 2. The HBR was larger when spatially congruent arrows were present compared to when there were incongruent arrows or no arrows. There was no difference between the HBR magnitude when incongruent arrows were fired and when no arrows were fired. *Bottom panel: The best-fitting geometric model*. Hit probability predicted by the best fitting model is shown in three conditions: with no arrows present (left), with arrows flying from the forward direction, as in Experiment 1 (middle) and with arrows flying from the forward-left direction, as in Experiment 2 (right). When arrows are present, the area of high hit probability expands in a direction corresponding to the trajectory of the arrows.

In Experiment 1 we found that when arrows are being fired towards the participant, the HBR-derived DPPS was different: the stimulus proximity-response function changed, and the region within which HBR magnitude was large expanded. This can be interpreted as an outward expansion of DPPS in the presence of arrows (Fig. 2; *Geometric Modelling*). Importantly, the differences in HBR amplitude between the ‘*Arrows’* and ‘*No Arrows’* conditions were not equal at each of the three hand positions, and were maximal in the Middle position. Thus, we did not observe a simple overall increase in HBR magnitude when arrows were fired, but a specific distance-dependent modulation.

This observation is, to the best of our knowledge, the only instance of a PPS related measure being modulated by movement of environmental objects separate from the stimulus used to derive its response field. Considering the results of Noel *et al*.^27^ is relevant: they first derived PPS size (i.e. the region within which the response field exceeds a certain value^15^) using reaction times to somatosensory stimuli while auditory stimuli moved towards participants. They then observed that the spatial features of such PPS are modulated by walking: the proximity-dependent fall-off of reaction times was more gradual when participants were walking forward while they were exposed to the optic flow consistent with their forward motion, interpreted as a forward expansion of the PPS. However, this finding was not due to the visual stimulation, as in a crucial control experiment they observed a similar PPS expansion when participants were walking on a treadmill but without the optic flow consistent with their forward motion^27^. Thus, the present result seems to be the first evidence that the movement of environmental visual stimuli (which are separate from those eliciting PPS responses) is able to change PPS features.

From Experiment 1 alone it is unclear whether the DPPS expansion is stereotyped, or sensitive to the trajectory of the moving objects; Experiment 1 could not distinguish between omnidirectional expansion (i.e. occurring in all directions), stereotyped unidirectional expansion (e.g. occurring only in front of the participant), and an expansion occurring only in the direction of the stimulus (i.e. only towards the source of the arrows). Therefore, in Experiment 2 we compared the effect of arrows with different trajectories. We observed that the HBR magnitude was increased only when the arrows’ trajectory was congruent to the stimulated hand, while the HBR magnitude was not modulated at all when the arrows’ trajectory was incongruent to the hand (Fig. 2; *Experiment 2*). These results provide strong evidence that the DPPS expansion is tuned to the direction of the source of the arrows.

Thus, the present result shows that the nervous system can intelligently respond to the specific dynamics of moving environmental objects. This is another example of a high-level factor, presumably occurring at neocortical level, modulating the HBR circuitry at brainstem level. Other examples include the probability of occurrence of the reflex-eliciting stimulus, the estimated protective value of objects, and the effects of gravity^12,21^. In all, these factors point towards a remarkably sophisticated mechanism which continuously adjusts the strength of motor defensive responses^15^, using information from multiple sensory modalities: proprioceptive (eye-hand proximity^8^), vestibular (gravitational cues^21^) and visual (moving arrows).

We have previously supplied evidence that this mechanism involves estimating the probability that threats will interact with, and thus damage, the face. In several studies, we showed that HBR results fit such a geometric model well, under a variety of hand positions and postural manipulations^14,21^. Here, we confirm that this model is sufficient to explain the observed changes in the HBR-derived DPPS, and add that it can also explain its directionally-tuned modulation. Indeed, in the model that fit the data of both Experiments 1 and 2, the presence of environmental moving objects (i.e. the arrows) biased the probability that the threat would hit the face. This model can therefore explain both the change in shape of the proximity-dependent function observed in Experiment 1, as well as the spatial congruence effect observed in Experiment 2. In Experiment 1, the increased bias towards the face altered the hit probability differently depending on the position of the hand, and hence increased the relevance of the HBR differently. In Experiment 2, when arrows were congruent to the stimulated hand, an increased bias in the direction of the arrows’ trajectory (i.e. towards the face) increased the estimates of hit probability, and hence increased the relevance of the HBR (Fig. 2; *Geometric Modelling*). However, when the arrows’ direction was incongruent the bias did not point towards the face, and thus did not increase the estimates of hit probability.

Thus, the present results reflect a clear directionally-tuned effect of arrows on the shape of the HBR-derived DPPS surrounding the face (Fig. 2; *Geometric Modelling*). But is this effect due to the fact that arrows are inherently threatening objects? Other PPS-related measures are clearly affected by the semantic content of stimuli^28,29^. Alternatively, is the present effect caused by the arrows’ speed? Or by their size and shape? Indeed, we already know that non-movement related factors such as trigeminal neuralgia (a condition in which innocuous trigeminal stimulation triggers paroxysmal facial pain) can affect HBR response fields^30^. As we were interested specifically in the effect of the *trajectory* of environmental objects, our experiment was not designed to investigate the contribution of other object features such as their semantics, speed, size and shape. We therefore have no relevant data to directly address this point. However, the congruence effect observed in Experiment 2 did show that the movement trajectory of environmental objects not related to the stimulus used to elicit the HBR was crucial to the DPPS expansion. Particularly, there was no increase of HBR magnitude in trials in which the arrows followed a trajectory that did not pass through the stimulated hand compared to the trials without arrows (Fig. 2; *Geometric Modelling*). Thus, it is unlikely that the shape or semantic information *alone* could have caused the observed effect; at most they were necessary but not sufficient causes. For example, it may be the case that arrows need to fly at a minimum speed to produce this effect, or that the looming object must be perceived as threatening (as in de Haan *et al*.^31^). Therefore, whether the effect we observed was only consequent to the moving trajectory, and that any object being fired towards the participants would modulate the HBR response field in the same direction-selective fashion remains an open question to be addressed in future studies.

Certain cortical areas in the primate brain have response profiles that could underlie the previously-described proximity-response relationship of the HBR^8,14,20^, as well as the additional directionally-sensitive modulation we report here. These include the ventral intraparietal area (VIP) and the polysensory zone of F4, which both contain bimodal neurons that respond to (1) tactile stimuli within a tactile receptive field, and (2) visual stimuli presented in a receptive field anchored to the tactile receptive field. Furthermore, many of these neurons are sensitive to combinations of joint angles and manipulations^32–36^. Additionally, neurons in these areas are sensitive to stimulus motion: for example, VIP neurons have a high degree of selectivity for the direction of stimulus movement and are also selective for stimulus speed^32^, while many neurons in F4 have a visual receptive field that expands with the velocity of approaching stimuli^33^. These areas also receive inputs from the superior colliculus and pulvinar^37^, both of which respond to looming stimuli and are involved in time-to-impact judgements^38^. We could therefore imagine a population of neurons with tactile receptive fields on the face, and visual receptive fields extending away from the face. The visual receptive field properties of such neurons could enact the movement sensitivity of the brainstem circuit subserving the HBR, while the joint-angle sensitivity of these neurons could enact the hand-position dependent manipulation of HBR magnitude. In contrast, the fact that the HBR modulation due to arrows is not equal at all hand positions (Fig. 2; *Experiment 1*) rules out the possibility that it was enacted by a multisensory neuron with a tactile receptive field on the wrist and a visual receptive field surrounding it: if this were the case, the HBR modulation should have been equal at all hand positions.

In conclusion, the DPPS derived from the HBR is sensitive to the ongoing movement and trajectory of other objects in the environment. This result, in conjunction with previous findings that this DPPS is affected by gravitational cues^21^ and physical barriers in the environment^12^, shows that it is not only sensitive to properties of the stimulus triggering the HBR, but also to the environmental context. These results support a view that the DPPS defined by a certain measure reflects the relevance of the action (or set of actions) linked to that measure^15^. Such relevance, which is partially determined by the probability that an object will make contact with the body, is therefore informed not only by the properties of the triggering stimulus, but also by the features of the environmental context which are relevant to impact prediction.

## Data Availability

Anonymised data is available on request.
